# Knocking Down TcNTPDase-1 Gene Reduces *in vitro* Infectivity of *Trypanosoma cruzi*

**DOI:** 10.3389/fmicb.2020.00434

**Published:** 2020-03-18

**Authors:** Natália Lins Silva-Gomes, Rita de Cássia Pontello Rampazzo, Claudia Maria do Nascimento Moreira, Gabriane Nascimento Porcino, Cyndia Mara Bezerra dos Santos, Marco Aurélio Krieger, Eveline Gomes Vasconcelos, Stenio Perdigão Fragoso, Otacilio C. Moreira

**Affiliations:** ^1^Laboratory of Molecular Biology and Endemic Diseases, Oswaldo Cruz Institute, Oswaldo Cruz Foundation, Rio de Janeiro, Brazil; ^2^Laboratory of Functional Genomics, Carlos Chagas Institute, Oswaldo Cruz Foundation, Curitiba, Brazil; ^3^Laboratory of Molecular Biology of Trypanosomatids, Carlos Chagas Institute, Curitiba, Brazil; ^4^Laboratory of Structure and Function of Proteins, Institute of Biological Sciences, Federal University of Juiz de Fora, Juiz de Fora, Brazil

**Keywords:** *Trypanosoma cruzi*, knockout, TcNTPDase-1, infectivity, virulence

## Abstract

Ecto-Nucleoside Triphosphate Diphosphohydrolases are enzymes that hydrolyze tri- and/or diphosphate nucleosides. Evidences pointed out to their participation in *Trypanosoma cruzi* virulence, infectivity, and purine acquisition. In this study, recombinant *T. cruzi* knocking out or overexpressing the TcNTPDase-1 gene were built, and the role of TcNTPDase-1 in the *in vitro* interaction with VERO cells was investigated. Results show that epimastigote forms of hemi-knockout parasites showed about 50% lower level of TcNTPDase-1 gene expression when compared to the wild type, while the *T. cruzi* overexpressing this gene reach 20 times higher gene expression. In trypomastigote forms, the same decreasing in TcNTPDase-1 gene expression was observed to the hemi-knockout parasites. The *in vitro* infection assays showed a reduction to 51.6 and 59.9% at the adhesion and to 25.2 and 26.4% at the endocytic indexes to the parasites knockout to one or other allele (Hygro and Neo hemi-knockouts), respectively. In contrast, the infection assays with *T. cruzi* overexpressing TcNTPDase-1 from the WT or Neo hemi-knockout parasites showed an opposite result, with the increasing to 287.7 and 271.1% at the adhesion and to 220.4 and 186.7% at the endocytic indexes, respectively. The parasitic load estimated in infected VERO cells by quantitative real time PCR corroborated these findings. Taken together, the partial silencing and overexpression of the TcNTPDase-1 gene generated viable parasites with low and high infectivity rates, respectively, corroborating that the enzyme encoded for this gene plays an important role to the *T. cruzi* infectivity.

## Introduction

Chagas disease is an endemic zoonosis in some countries of Central and South America that has *Trypanosoma cruzi* as its etiological agent, which affects 6–7 million individuals and still remains a major public health problem (World Health Organization [WHO], 2017). *T. cruzi* is represented by a group of isolates that shows distinct levels of sensitivity to drugs, disease prognosis and pathogenicity (Macedo and Pena, 1998; Campbell et al., 2004), as well as eco-epidemiological complexity (Miles et al., 2009; Brenière et al., 2016; Rodrigues-dos-Santos et al., 2018). The chemotherapy currently used is based on antiparasitics such as, nifurtimox and benznidazole, which cause many side effects and present a lack of effectiveness on the chronic phase of the disease (Bern, 2011; Morillo et al., 2015). In this scenario, the search for new drugs and targets to chemotherapy is pivotal. In particular, molecules localized at the parasite plasma membrane surface appear to be better targets, since they could be more exposed to the drugs.

Nucleoside triphosphate diphosphohydrolases (NTPDase; EC 3.6.1.5) are ubiquitously distributed glycoproteins that hydrolyze tri- and diphosphate nucleosides to the monophosphate form. They are characterized by the presence of five apyrase conserved regions (ACR1 to ACR5) and, in conjunction with the nucleotidases, these enzymes are capable to generate phosphate-free nucleosides. The ecto-NTPDases class consists in two main types of enzymes: ATP diphosphohydrolase (which hydrolyze both ATP and ADP, also known as ATPDase or apyrase) and the ecto-ATPases (Plesner, 1995; Zimmermann, 1999). These enzymes have multiple roles associated with *T. cruzi* virulence such as ability of the parasites to regulate the cell signaling triggered by extracellular ATP and other nucleotides (Silverman et al., 1998; Sansom et al., 2007), that are generated during the lysis of the *T. cruzi*-infected cells (Schnurr et al., 2000; Sansom et al., 2007). Since extracellular ATP is an immune-modulatory molecule that in normal conditions stimulates the secretion of proinflammatory cytokines (IFN-ɤ and IL-2) to control the infection (Langston et al., 2003), it is hypothesized that ATP hydrolysis by ecto-ATPases activity in parasites can be important to subvert and avoid host defense mechanism, although the mechanism is not clearly elucidated (Sansom et al., 2008). Another important role of these enzymes is in parasite nutrition, facilitating acquisition of extracellular purines since these parasites are not capable for performing *de novo* synthesis.

The role of NTPDases in the processes of infectivity and virulence of parasites has been explored. In *Toxoplasma gondii*, it has been demonstrated that host cells treated with dithiols, which increase ecto-ATPase activity of a secreted NTPDase, have depletion of ATP levels and mass exodus of the intracellular parasites suggesting an important role in the parasite exit from the cell (Silverman et al., 1998; Carruthers, 1999). Treatment of the distinct evolutive forms of *T. cruzi* with DIDS and Suramin lead to ecto-ATPase activity inhibition, and *in vitro* reductions in parasite adhesion and internalization in the macrophages. In addition, the increasing of ecto-ATPase activity was followed by a parallel increasing in parasite adhesion to resident macrophages (Bisaggio et al., 2003). More recently, by the use of anti-TcNTPDase-1 polyclonal antibodies as blockers, or recombinant TcNTPDase-1 as a competitor, *in vitro* inhibition of parasite infection was observed, suggesting that this enzyme has a role in parasite–host interaction and cell adhesion (Mariotini-Moura et al., 2014).

Nevertheless, the knowledge about the role of NTPDases in parasites still has many gaps. Most studies published so far have focused on the enzymatic activity of ecto-NTPDases in living parasites or plasma membrane fractions. Considering that different enzymes or isoforms with nucleotidase activity can be located at *T. cruzi* plasma membrane, a molecular approach could improve the specificity for the analysis of NTPDase-associated genes role to the infectivity and virulence of protozoan parasites. Thus, in this study, recombinant *T. cruzi* (Dm28c clone, Tc I) knocking-down or overexpressing the TcNTPDase-1 gene (AY540630.1) were generated, in order to evaluate the specific contribution of this enzyme in the parasite infectivity, using an *in vitro* model of VERO cells infection with metacyclic recombinant trypomastigotes.

## Materials and Methods

### Parasite Cultivation

*Trypanosoma cruzi* epimastigotes (Dm28c clone) were cultured in LIT medium supplemented with 10% heat-inactivated bovine fetal serum (Invitrogen) at 28°C for 5 days, to reach log-growth phase. To obtain metacyclic trypomastigotes, *T. cruzi* were allowed differentiated under chemically defined conditions (TAU3AAG medium), as previously described (Contreras et al., 1985; Bonaldo et al., 1988). Briefly, epimastigotes in the late exponential growth phase were harvested from LIT medium by centrifugation and subjected to nutritional stress for 2 h in triatomine artificial urine (TAU, 190 mM NaCl, 17 mM KCl, 2 mM MgCl_2_, 2 mM CaCl_2_, 8 mM sodium phosphate buffer, pH 6.0) at a density of 5 × 10^8^ cells/mL. They were then transferred to cell culture flasks containing TAU3AAG (TAU supplemented with 50 mM sodium glutamate, 10 mM L-proline, 2 mM sodium aspartate, 10 mM glucose, at a density of 5 × 10^6^ cells/mL at 28°C). After 72 h of incubation, metacyclic trypomastigotes were obtained.

To obtain cell-derived trypomastigotes, metacyclic trypomastigotes were collected as described above and used to infect VERO cells, that were growth in RPMI medium supplemented with 5% heat-inactivated bovine fetal serum, 100 U/mL penicillin, 10 μg/mL streptomycin and 2 mM L-glutamine at 37°C in an atmosphere of 5% CO_2_. The cell monolayer was then infected with metacyclic trypomastigotes (10 parasites for each host cell). After 24 h, the medium was discarded to remove non-internalized parasites. Cells were then washed once with PBS and new medium was added to the culture flasks. Cells-derived trypomastigotes were released into the supernatant 3 days after infection and were harvested by centrifugation at 3500 rpm for 10 min.

### Knockout and Overexpression of the TcNTPDase-1 Gene

The selectable markers NEO and HYGRO encoding resistance to G418 and hygromycin B, respectively, were amplified by PCR and inserted into pBluescript SKII(+) plasmid (Stratagene). The NEO gene was inserted between the *Sal*I and *Eco*RI sites, whereas the HYGRO gene was inserted between the *Xba*I and *Bam*HI sites. The recombinant plasmids were named pTc2KO-neo and pTc2KO-higro, respectively.

A 761-bp fragment of the coding region of the NTPDASE-1 gene was amplified by PCR with the CDS_*Kpn*I Fw (5′-ATGC**GGTACC**CCTTGCGCTGCTCTGCCTCTTTC-3′) and CDS_*Sal*I Rv (5′-GGTA**GTCGAC**CAGGGGTGAAAGGGATGC GA-3′) primers from the *T. cruzi* (clone Dm28c) genomic DNA. Similarly, a fragment of the downstream intergenic region of the NTPDASE-1 gene was amplified with primers DOWN_*Bam*HI Fw (5′-GCAT**GGATCC**GCACTTGGCGCCTCCCTTGTTA-3′) and DOWN_*Xba*I Rv (5′-GGAG**TCTAGA**AATTCCCCGCA CTTTCACCTCCC-3′). The PCR products, 761 and 641, respectively, were purified using Qiaquick PCR purification (Qiagen), digested with *Kpn*I and *Sal*I (CDS fragments) and digested with *Bam*HI e *Xba*I (downstream fragments) and were sequentially inserted into the pTc2KO-neo. The recombinant plasmid was named pTc2KO-NTPDASE-neo.

A 448-bp upstream fragment of NTPDASE-1 gene was amplified by PCR with primers UPS_*Kpn*I Fw (5′-GATC**GGTACC**CCAATGCGAATCCACATTGCGGTGGTTTT CGGTCGTGT-3′) and UPS_*Sal*I (5′-GGGG**GTCGAC**CGCCAC TGAACTCCGCCGTGATAATGT-3′) from *T. cruzi* (clone Dm28c) genomic DNA. Similarly, a fragment of the downstream intergenic region of the NTPDASE-1 gene was amplified with primers DOWN_*Bam*HI Fw (5′-GCAT**GGATCC**GCACTTGGCGCCTCCCTTGTTA-3′) and DOWN_*Xba*I Rv (5′-GGAG**TCTAGA**AATTCCCCGCACT TTCACCTCCC-3′). The PCR products, 448 and 641, respectively, were purified using Qiaquick PCR purification (Qiagen), digested with *Kpn*I and *Sal*I (upstream fragments) and digested with *Bam*HI e *Xba*I (downstream fragments) and were sequentially inserted into the pTc2KO-higro. The recombinant plasmid was named pTc2KO-NTPDASE-hyg.

The recombinant plasmids pTc2KO-NTPDASE-neo and pTc2KO-NTPDASE-hyg were purified by the alkaline lysis method using the plasmid mini-preparation Kit (Qiagen). The minipreps were used for amplification of the CDS/NEO/DOWN region (NEO cassete) and the UPS/HYG/DOWN region (HYG cassete) by PCR, using CDS_*Kpn*I (foward) and DOWN_*Xba*I (reverse) primers for the NEO cassete and UPS_*Kpn*I (foward) and DOWN_*Xba*I (reverse) for the HYG cassete. The amplified material was purified by phenol/chloroform extraction, followed by absolute ethanol precipitation (Medina-Acosta and Cross, 1993). A total of 25 μg of NEO and HYG cassetes DNA, separately, were used to transfect *T. cruzi*, based on previously described method (Lu and Buck, 1991). The GenePulser II Electroporator (Bio-Rad) was used to transfect 1 × 10^8^ cells in 0.2 cm cuvettes; cells were treated with two 450V/500 μF pulses. After 24 h, 500 μg/mL of G418 (Sigma Aldrich) or hygromycin B (Sigma Aldrich) were added to select the transfected parasites. Transfectants were cloned by serial dilution in 24-well plates. Isolates were analyzed for the correct insertion of the NEO and HYG genes into the locus of NTPDASE1. Null mutants were maintained in LIT medium for at least 20 passages in the presence of the antibiotics, with each passage lasting a week.

In order to perform a reverse test of the results obtained with knockout parasites, genetically modified organisms overexpressing the TcNTPDase-1 gene were also produced. Thus, the integrative vector pBEX v2.0 was used, modified from the initial version as previously described (Kessler et al., 2013). The NTPDASE-1 gene was amplified with the primers NTPDASE_*Bam*HI_f (5′-GGGGGGATCCATGAAGCAGAGCATGGCACG-3′) and NTP- DASE_*Sal*I_r (5′-GGGGGTCGACTTAAGCAGATTGTCCCT CTAAACTAACAAGG-3′) and cloned into the vector pBEX v2.0 at the *Bam*HI and *Sal*I sites. Identification of the positive clones and preparation of the plasmids for transfection of *T. cruzi* was performed as described above. For transfection, epimastigote forms of *T. cruzi* were cultured in LIT medium to a density of 2 × 10^7^ cells/mL. The parasites (2 × 10^8^ cells) were collected by centrifugation at 3000 *g* for 10 min at 4°C. The cell pellet was washed with sterile PBS and ressuspended in 1 mL electroporation solution (140 mM NaCl, 25 mM Hepes (acid) and 0.74 mM Na_2_HPO_4_, pH 7.5). Volumes corresponding to 0.4 mL of the cell suspension were transferred to two sterile electroporation cuvettes (0.2 cm GAP) (Bio-Rad), and 10 μg of plasmid pBEX/NTPDASE-1 was added in one. The other bucket containing only the parasite suspension was used as control. After 10 min on ice, the samples contained in the cuvettes were subjected to two pulses of 450 V/500 μF using the Gene Pulser II Apparatus (Bio-Rad) electroporator. Samples were incubated for 5 min at room temperature and then transferred to 25 cm^2^ culture bottles containing 10 mL of LIT medium supplemented with 10.000 U of penincilin and 10 μg/mL of streptomycin. The cultures were then incubated at 28°C. After 24 h of incubation, antibiotic G418 (Sigma Aldrich) at the concentration of 500 μg/mL was added. Cultures were maintained by successive passages (1:10 dilution) in LIT media supplemented with G418 every 8–10 days, until no cell proliferation in the control culture. Protein extracts from the G418-resistant parasites were tested by Western blot for the overexpression of NTPDase-1.

### *T. cruzi* Mammalian Cell Adhesion and Invasion Assays

Trypomastigote forms of *T. cruzi* Dm28c strain were obtained from the supernatant of previously infected VERO cells grown in RPMI medium (Gibco) in a humidified 5% CO_2_ atmosphere. *In vitro* host cell assays were carried out as detailed elsewhere (Santos et al., 2009), using second passage trypomastigotes (P2). Briefly, 2 × 10^5^ trypomastigotes from Dm28c strain were placed in each well of 24-well plates containing 13 mm round glass cover slips coated with 2 × 10^4^ VERO cells (10:1). For cell adhesion assay, after 2 h of interaction, the cover slips were gently washed three times with PBS, fixed and stained with Panotic Kit (NewProv). The number of cells containing adhered parasites and the number of parasites per cell were counted in at least 200 cells, in triplicate, by light microscopy. For cell invasion assays, after 48 h of infection, the numbers of infected cells and amastigotes per infected cell were counted under the same experimental condition. The adhesion index was obtained by multiplying the percentage of VERO cells with adhered parasites by the average of adhered parasites per VERO cell ratio. The endocytic index was obtained by multiplying the percentage of infected VERO cells by the average of the amastigotes per infected VERO cells ratio.

### RNA Isolation and cDNA Synthesis

Total RNA from *T. cruzi* (1 × 10^8^ cells) was extracted using TRIzol Reagent (Invitrogen) and treated with DNAse I (Sigma Aldrich), following manufacturer’s instructions. RNA quantity and purity were estimated by spectrophotometry at 260/280/230 nm. RNA integrity was verified through electrophoresis on a 1.5% (w/v) agarose gel. All reverse transcriptase reactions were performed from 3 μg of RNA using a Superscript III First-strand System (Invitrogen), according to the manufacturer’s instructions.

### NTPDase-1 Gene Expression Quantification by Real Time RT-PCR

Real-time quantitative PCR assays were performed in ABI Prism 7500 fast sequence detection system using Power SYBR Green PCR Mastermix (Applied Biosystems). The following primers and concentrations were used: TcNTPDase-1 Fw (300 nmol/L), 5′-GCACGCTGCTAAGGAACAAC-3′; TcNTPDase-1 Rv (300 nmol/L), 5′-TCTTGGACCTTGGAGTTCGC-3′; TcCalmoduline Fw (600 nmol/L), 5′-CCCGACGGAGGCGGAGCTGC-3′; TcCalmoduline Rv (600 nmol/L), 5′-GTCCACGTCGGCC TCGCGGA-3′; TcGAPDH Fw (300 nmol/L), 5′-GTGCGG CTGCTGTCAACAT-3′; andTcGAPDH Rv (300 nmol/L), 5′-AAAGACATGCCCGTCAGCTT-3′. The conditions for the RT-qPCR were as follows: 95°C for 10 min, followed by 40 cycles at 95°C for 15 s and 62°C for 1 min. To monitor the primers specificity, melting curves were performed after each experiment, resulting in a single peak. Reactions were performed in duplicates using 2 μL of cDNA template, in a total volume of 20 μL. The relative quantitative measurement of target gene levels was performed using the ΔΔCt method (Livak and Schmittgen, 2001). As endogenous housekeeping control genes, *T. cruzi* Calmoduline and GAPDH genes were used. PCR assays were in triplicate and data were pooled.

### Parasite Load Quantification in VERO Cells by Quantitative Real-Time PCR (qPCR)

DNA was extracted from the infected VERO cells monolayer using the TRIzol reagent (Sigma Aldrich), according to the manufacture’s instruction. Then, 5 μL of DNA were analyzed by a quantitative real-time multiplex PCR assay. The absolute quantification of parasite load was performed in an Applied Biosystems 7500 Fast Real-Time PCR instrument, using primers Cruzi 1 (5′-ASTCGGCTGATCGTTTTCGA-3′), Cruzi 2 (5′-AATTCCTCCAAGCAGCGGATA-3′) and probe Cruzi 3 (5′-FAM-CACACACTG GACACCAA-NFQ-MGB-3′) targeting *T. cruzi* nuclear satellite DNA and an internal amplification control (IAC; plasmid pZErO-2 containing an insert from the *Arabidopsis thaliana* aquaporin gene), with primers IAC *Fw* (5′-ACCGTCATGGAACAGCACGTA-3′), IAC *Rv* (5′-CTCCCGCAACAAACCCTATAAAT-3′) and probe IAC (5′-VIC-AGCATCTGTTCTTGAAGGT-NFQ-MGB-3′), as previously described (Duffy et al., 2013). A standard curve was generated by a 1:10 serial dilution of DNA extracted from epimastigote culture stocks of the *T. cruzi* Dm28c strain, ranging from 10^6^ to 0.5 parasite equivalents. The conditions for the qPCR were as follows: 50°C for 5 min, 95°C for 10 min followed by 40 cycles at 95°C for 15 s and 58°C for 1 min. The qPCR assays were performed in duplicate for each sample, and three biological replicates were analyzed.

### Polyacrylamide Gel Electrophoresis and Western Blots

Parasites were grown in LIT medium supplemented with 10% of bovine fetal serum for 5 days. After that, 10^8^ parasites were washed three times in sterile PBS by centrifugation at 3500 *g* for 10 min, at 4°C. The cells were submitted to five freeze-thaw cycles and a protease inhibitor cocktail was added (Sigma Aldrich). Aliquots of this epimastigotes preparation (100 μg of total protein) were dissolved in gel loading buffer, and submitted to sodium dodecyl sulfate-10% polyacrylamide gel electrophoresis (SDS-PAGE), using Mini-Protean III Cell (Bio-Rad). The proteins were electroblotted onto nitrocellulose membranes, followed by blocking step (0.15M phosphate buffer solution, pH 7.4, plus 0.3% Tween-20 and 2% casein) using standard procedures. Rabbit serum containing polyclonal antibodies against potato apyrase that have cross-immunoreactivity with NTPDase 1 from *T. cruzi* epimastigote (Faria-Pinto et al., 2008) was diluted (1:5000) in the same blocking buffer without Tween-20 and incubated overnight. Signals were revealed by chemiluminescence using anti-rabbit IgG coupled to horseradish peroxidase and luminol as substrate (GE Healthcare) and exposed to X-ray film following the manufacturer’s instructions. As loading control, the anti-tubulin (1:1.000) was used as primary antibody, and anti-mouse IgG coupled to horseradish peroxidase as secondary antibody, using the same experimental procedures. Densitometric analysis was performed with the Quantity One (Bio-Rad).

### Statistical Analysis

All experiments were performed at least in biological triplicates and experimental duplicates. Data are expressed as arithmetic mean ± standard deviation. Student’s *t*-test or Mann–Whitney Rank-Sum test were adopted to analyze the statistical significance of the apparent differences. All statistical tests were performed with SigmaPlot for Windows Version 12 (Systat Software). Differences were considered statistically significant when *p* < 0.05.

## Results

### *Trypanosoma cruzi* NTPDase-1 Gene Knockout Strategy

The diagrams at Supplementary Figures S1A,C show the strategy used for the integration of the cassettes to confer resistance to Hygromycin (HYGRO) and Neomycin (NEO), respectively, at the locus of the *T. cruzi* NTPDase-1 gene. In both cases, the cassette integration disrupts the TcNTPDase-1 CDS in each allele, promoting the knocking down of the TcNTPDase-1 expression. After the selection of the hygromycin or neomycin resistant-parasites, the validation of the cassette integration at the expected position was performed by PCR. In Supplementary Figures S1B,D, PCR products that confirm the expected integration of the HYGRO and NEO cassettes in the correct position are shown, including the upstream intergenic region amplification with 769/877 and 1646/1754 bp to the HYGRO and NEO cassettes, respectively, and the downstream intergenic region amplification with 887/1027 bp to both cassettes.

### Growth Curve of Hemi-Knockouts for TcNTPDase-1

Once confirmed the insertion of the resistance gene into the TcNTPDase-1 genomic locus and established the resistance of these parasites to neomycin and hygromycin, parasites were separated through cell sorting, in four clones for each hemi-knockout: Hygro (#1), (#2), (#3), and (#4) and Neo (#1), (#2), (#3), and (#4). These clones were monitored during all phases of cell cultivation, in comparison to the wild type (WT) parasite. Thus, epimastigotes were cultured in LIT medium up to 10 days, at 28°C.

**FIGURE 1:**
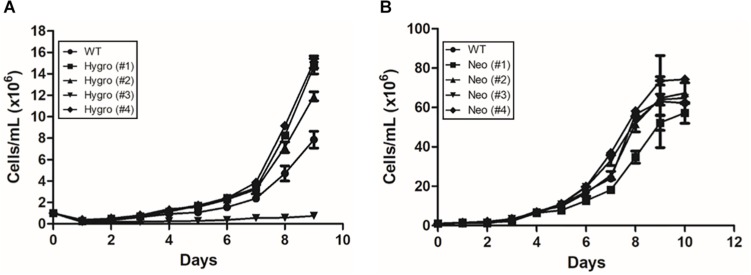
Growth curve of hemi-knockout epimastigote clones in LIT medium at 28°C, up to 10 days of cultivation. **(A)** Hygromycin-resistant hemi-knockout clones. (●) Dm28c WT; (■) Hygro (#1); (▲) Hygro (#2); (▼) Hygro (#3); (■) Hygro (#4). **(B)** Neomicyn-resistant hemi-knockout clones. (●) Dm28c Wt; (■) Neo (#1); (▲) Neo (#2); (▼) Neo (#3); (■) Neo (#4).

As observed in Figure 1A, the hygromycin-resistant hemi-knockout clones Hygro (#1), (#2), and (#4) presented a slightly higher, but not statistically significant, growth rate when compared to the WT clone. On the other hand, the clone Hygro (#3) presented a lower growth rate when compared to the control (Figure 1A). For neomycin-resistant hemi-knockout clones (Figure 1B), there was no significant difference between the cellular growth rate among them, although the Neo (#1) was the one with the lowest growth rate.

### TcNTPDase-1 Gene Expression in Non-infective and Infective Forms of *T. cruzi* Hemi-Knockouts

**FIGURE 2:**
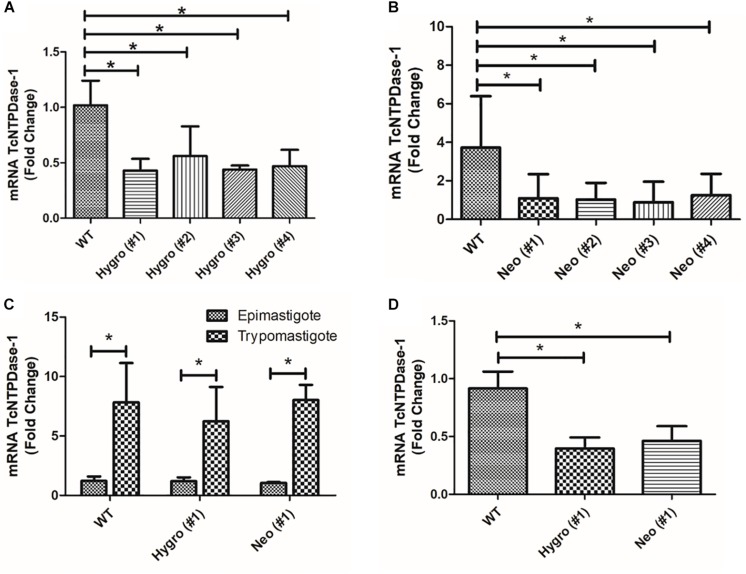
TcNTPDase-1 gene expression in distinct evolutive forms of *T. cruzi* hemi-knockouts. **(A,B)** TcNTPDase-1 mRNA levels in epimastigote forms of HYGRO and NEO hemi-knockout clones, respectively. The relative quantification by ΔΔCt method was performed using the wild type (WT) clone as the calibrator. **(C)** TcNTPDase-1 mRNA levels in trypomastigote forms of WT, HYGRO, and NEO hemi-knockouts. Epimastigote forms of the respective clones were used as calibrators. **(D)** TcNTPDase-1 mRNA levels in trypomastigote forms of the hemi-knockouts (HYGRO and NEO) in comparison to the WT clone. Trypomastigote forms of the WT clone were used as the calibrator. *p < 0.05 (Student’s *t*-test).

In order to validate if the TcNTPDase-1 gene expression in the hemi-knockouts would be reduced in epimastigote forms of *T. cruzi*, TcNTPDase-1 mRNA levels were quantified in comparison to WT clone by the comparative Ct method (ΔΔCt) (Livak and Schmittgen, 2001). In the Figures 2A,B, significant decreases in the TcNTPDase-1 gene expression between Hygro clones (**#1** = 0.4 ± 0.1; **#2** = 0.6 ± 0.3; **#3** = 0.4; **#4** = 0.5 ± 0.1) and Neo clones (**#1** = 1.1 ± 2.7; **#2** = 1.0 ± 0.9; **#3** = 0.9 ± 1.1; **#4** = 1.2 ± 1.1) when compared to the respective WT parasites (1.0 ± 0.2 and 3.7 ± 2.7, respectively) were observed. It also was evaluate if the TcNTPDase-1 expression could be reduced in trypomastigote forms of *T. cruzi* hemi-knockouts. For this, cell-derived trypomastigote forms were obtained to Hygro (#1), Neo (#1) and the WT clones, after infection of VERO cells using metacyclic trypomastigotes. In Figure 2C, it is possible to observe that trypomastigote forms of WT, Hygro (#1) and Neo (#1) showed significant increases of 7.8 ± 3.3, 6.2 ± 2.9, and 8.0 ± 1.3 times in the expression of TcNTPDase-1 in comparison to their respective epimastigote forms. However, mRNA levels of the trypomastigotes Hygro (#1) and Neo (#1) were around 60 and 50% lower (Figure 2D), respectively, than the trypomastigotes obtained from the WT clone, indicating that the hemi-knockouts also maintain TcNTPDase-1 gene expression level significantly lower in the trypomastigote forms of the parasite.

### Analysis of TcNTPDase-1 Protein Levels by Western Blotting

**FIGURE 3:**
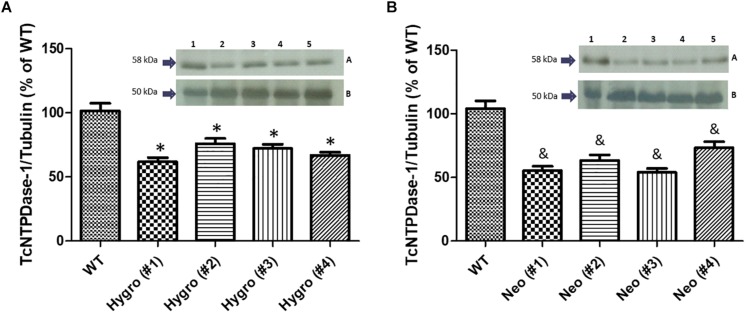
Estimation of TcNTPDase-1 protein levels in hemi-knockout epimastigote clones by Western blots. **(A)** HYGRO hemi-knockout epimastigote clones. **(B)** NEO hemi-knockout epimastigote clones. 1: WT; 2: (#1); 3: (#2); 4: (#3); 5: (#4). Insets: **(A)** Anti-potato apyrase serum (1:5000). **(B)** Anti-tubulin antibodies (1:1000). ^∗^,^&^*p* < 0.05 (Student’s *t*-test).

In order to evaluate if the observed decrease in mRNA levels for the hemi-knockout parasites would also result in a decrease of the correspondent TcNTPDase-1 protein levels, Western blot assays were performed using hemi-knockout epimastigote protein extracts and anti-potato apyrase serum, which detects the *T. cruzi* NTPDase-1 by cross-immunoreactivity. Anti-*T. cruzi* tubulin antibodies were used as loading control. As shown in Figure 3A, mild but significant reductions in TcNTPDase-1 expression levels were observed for HYGRO clones (WT = 101.5 ± 10.5% **#1** = 61.8 ± 5.6%; **#2** = 76.0 ± 7.1%; **#3** = 72.1 ± 5.8%, and **#4** = 66.9 ± 4.2%). In contrast, there was higher and also significant decrease in TcNTPDase-1 expression levels for the NEO clones (Figure 3B, WT = 104.0 ± 10.7% **#1** = 55.3 ± 6.0%; **#2** = 63.2 ± 7.9%; **#3** = 54.0 ± 5.4%, and **#4** = 73.3 ± 8.5%).

### Experimental Infection in VERO Cells

#### Effect of TcNTPDase-1 Hemi-Knockout in *T. cruzi* Adhesion to VERO Cells

**FIGURE 4:**
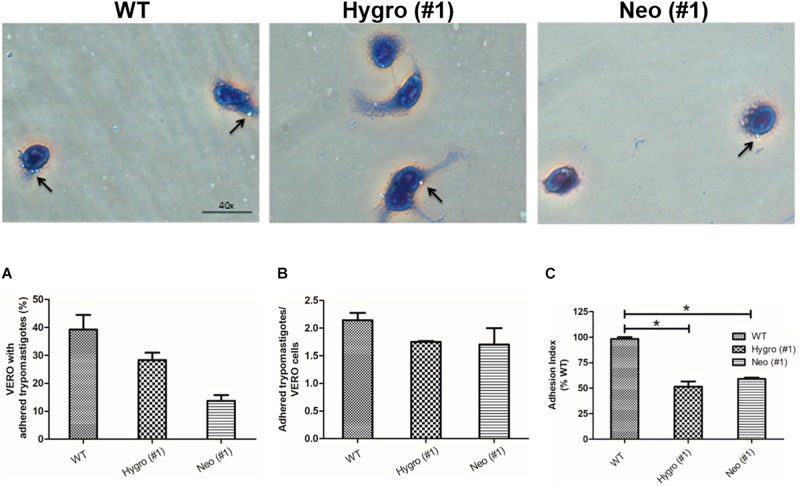
Adhesion assay of hemi-knockout parasites to VERO cells. The adhesion of WT, HYGRO, and NEO hemi-knockout trypomastigotes to VERO cells were estimated after 2 h of interaction. **(A)** Percentage of VERO cells with adhered trypomastigotes. **(B)** Number of trypomastigotes adhered per VERO cells. **(C)** Adhesion index. The asterisks show significant differences (*p < 0.05, Student’s *t*-test) between the control and hemi-knockout parasites. At the top, representative light microscopy images show VERO cells with adhered trypomastigotes. The arrows indicate adhered trypomastigotes and the scale bars correspond to 40 μm.

To understand the role of *T. cruzi* TcNTPDase-1 in the adhesion process to mammalian cells, *in vitro* infections in VERO cells were performed using metacyclic trypomastigotes forms of the hemi-knockout clones HYGRO (#1), NEO (#1) and WT. After 2 h of interaction, the numbers of cells containing trypomastigotes forms adhered and adhered trypomastigotes per cell were counted (Figure 4, top). As shown in Figure 4A, significant reduction of adhered parasites to the Vero cells was observed. WT clone presented an cell adhesion rate of 39.2 ± 9.1%, while those promoted by the clones HYGRO (#1) and NEO (#1) in VERO cells were only 28.3 ± 4.6 and 13.7 ± 2.9%, respectively (Figure 4A). However, only slight reductions were noted when the number of trypomastigotes per infected VERO cells was counted: from 2.1 ± 0.2 to 1.7 ± 0.1 and 1.7 ± 0.4 in the infections promoted by WT, HYGRO (#1) and NEO (#1), respectively (Figure 4B). As a consequence of this parasite–host interaction, the adhesion index was significantly reduced to 51.6 ± 8.7 and 59 ± 1.9% when infections were performed with the hemi-knockouts HYGRO (#1) and NEO (#1) clones, respectively, in comparison to the WT clone (Figure 4C).

#### Effect of TcNTPDase-1 Hemi-Knockout in *T. cruzi* Infection in VERO Cells

**FIGURE 5:**
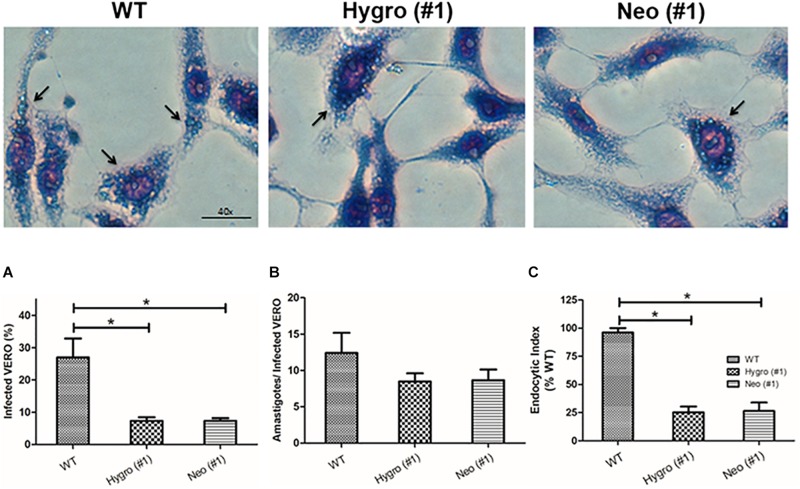
Infection assay of hemi-knockout parasites to VERO cells. The internalization of WT, HYGRO, and NEO hemi-knockout trypomastigotes to VERO cells were estimated after 48 h of interaction. **(A)** Percentage of VERO cells infected. **(B)** Number of intracellular amastigotes per VERO cells. **(C)** Endocytic index. The asterisks show significant differences (*p < 0.05, Student’s *t*-test) between the control and hemi-knockout parasites. At the top, representative light microscopy images show infected VERO cells. The arrows indicate amastigotes and the scale bars correspond to 40 μm.

In order to understand the role of TcNTPDase-1 in the internalization process of *T. cruzi* to mammalian cells, *in vitro* infections in VERO cells were performed using metacyclic trypomastigotes forms of the hemi-knockout clones HYGRO (#1), NEO (#1) and WT. After 48 h of infection, cells were analyzed by the percentage of infected cells and the number of amastigotes per cell (Figure 5, top). Figure 5A, shows the percentage of infected VERO cells that had internalized parasites (amastigote forms), where a significant decreasing was observed for infections with the hemi-knockout parasites. The WT parasite led to a rate of 26.9 ± 10.2% infected cells while in the infections promoted by the HYGRO (#1) and NEO (#1) clones, only 7.3 ± 1.9 and 7.3 ± 1.4%, respectively, of VERO cells containing amastigotes were observed. However, lower decreasing was observed when the number of amastigotes per infected VERO cell was counted: from 12.4 ± 4.8 to 8.5 ± 2.0 and 8.7 ± 2.5, to infections promoted by WT, HYGRO (#1) and NEO (#1), respectively (Figure 5B). As a consequence of this parasite–host interaction, the endocytic index was significantly reduced to 25.2 ± 9.2 and 26.4 ± 12.8% when infections were performed with the hemi-knockouts HYGRO (#1) and NEO (#1) clones, respectively, in comparison to the WT clone (Figure 5C).

#### Parasitic Load Quantification in VERO Cells Infected With TcNTPDase-1 Hemi-Knockouts

**FIGURE 6:**
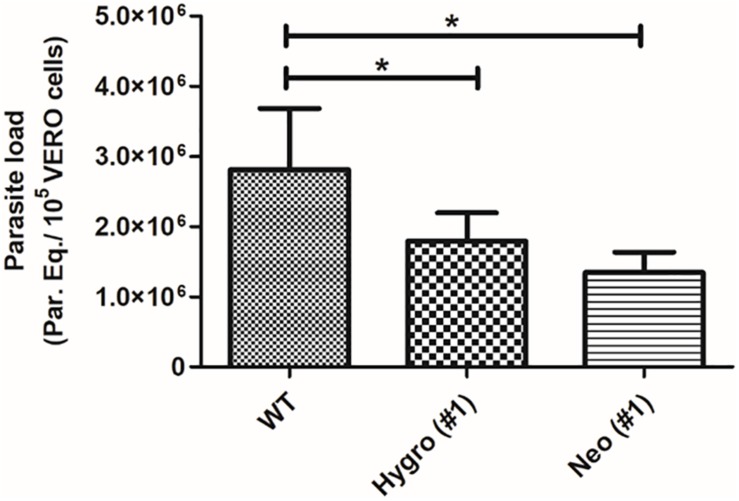
Parasitic load quantification in VERO cells infected with TcNTPDase-1 hemi-knockouts. VERO cells were infected for 48 h with metacyclic trypomastigotes of WT, HYGRO, and NEO hemi-knockout clones. DNA was extracted and parasitic load was estimated by real-time PCR. *p < 0.05 (Student’s *t*-test).

To confirm the decreasing of infectivity to TcNTPDase-1 hemi-knockouts in the VERO cells, the parasitic load resulting from the *in vitro* infection was quantified by Real-time PCR. Supplementary Figure S2 shows the dynamic range for the absolute quantification, with linearity from 10^6^ to 1 parasite equivalent. The generated standard curve shows an efficiency of 102.7% and a coefficient of determination (r^2^) of 0.99, indicating the high sensitivity and precision for the quantification. The parasitic load quantification to the infection promoted by WT, HYGRO, and NEO clones in VERO cells is observed in Figure 6. Accordingly, a reduced parasitic load was observed to the HYGRO (#1) and NEO (#1) hemi-knockout clones (1.8 and 1.3 Parasite equivalents/10^5^ VERO cells, respectively) in comparison to the WT clone (2.8 Parasite equivalents/10^5^ VERO cells).

### Reversing Knockout of the TcNTPDase-1 Gene

**FIGURE 7:**
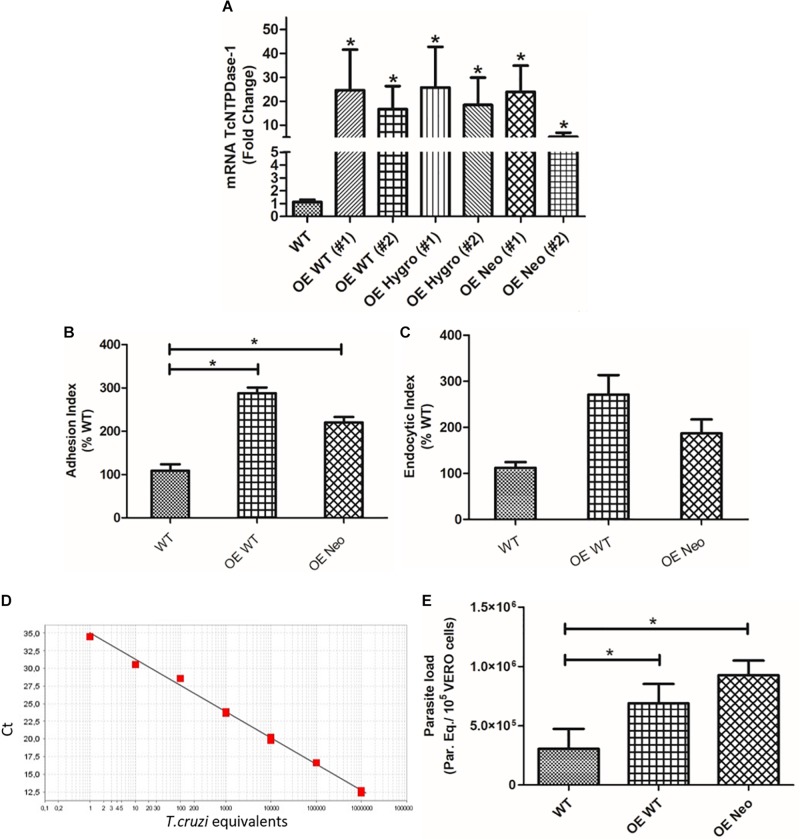
Overexpression of TcNTPDase-1 gene from WT or hemi-knockout parasites. **(A)** TcNTPDase-1 mRNA levels were estimated by RT-qPCR in WT and parasites overexpressing the gene. The relative quantification by ΔΔCt method was performed using the WT clone as the calibrator. *p < 0.05. **(B)** Adhesion index to the *in vitro* infection of VERO cells using the parasites overexpressing TcNTPDase-1. ^∗^*p* < 0.05. **(C)** Endocytic index to the *in vitro* infection of VERO cells with the parasites overexpressing TcNTPDase-1. **(D,E)** Parasitic load quantification in VERO cells infected with parasites overexpressing TcNTPDase-1 gene. VERO cells were infected for 48 h with metacyclic trypomastigotes of WT, OE WT, and OE Neo clones. DNA was extracted and parasitic load was estimated by real-time PCR. **(D)** Standard curve to the absolute quantification experiments. **(E)** Parasitic load in VERO Cells infected by WT and parasites overexpressing TcNTPDase-1. *p < 0.05 (Student’s *t*-test).

In order to confirm if the observed decrease in both gene expression, adhesion and endocytic indexes of hemi-knockout parasites would be, in fact, due to the partial silencing of the TcNTPDase-1 gene, we followed the reverse path. Thus, WT and hemi-knockout parasites were genetically modified to overexpress the TcNTPDase-1 gene. As showed in the Figure 7A, the TcNTPDase-1 gene expression in the overexpressing parasites, modified from the WT [OE WT (#1) and (#2)] or from the hemi-knockout [OE Hygro and OE Neo, (#1) and (#2)] were significantly increased (21.9 ± 12.0, 16.0 ± 6.8, 23.0 ± 12.0, 16.4 ± 8.1, 21.3 ± 7.7, and 4.6 ± 1.2 times higher, respectively) when compared to the WT clone Dm28c. Regarding the *in vitro* assays using VERO cells, both the adhesion and endocytic index increased when infections were performed using the OE WT and OE Neo (Figures 7B,C; 287.7 ± 13.13 and 271.1 ± 42.50 to the adhesion index and 220.4 ± 12.47 and 186.7 ± 30.28 to the endocytic index, respectively). Thus, in order to confirm the increased infectivity of the parasites overexpressing TcNTPDase-1 gene, parasitic loads in infected VERO cells were also quantified by Real-time PCR (Figures 7D,E). To the cells infected with the WT parasites, the parasitic load was 3.0 Parasite equivalents/10^5^ VERO cells. In contrast, significant increasing in the parasitic loads of cells infected with OE WT (6.9 Parasite equivalents/10^5^ VERO cells) and OE Neo (9.3 Parasite equivalents/10^5^ VERO cells) were observed, showing the reversal of the low-infectivity phenotype previously observed to the TcNTPDase-1 hemi-knockout parasites.

## Discussion

The external face of plasma membrane share both millimolar divalent cation-dependent ecto-ATPase and ecto-NTPDase activities. Ecto-ATPases hydrolyze ATP better than other trinucleotides while ADP is hardly hydrolyzed. On the other hand, ecto-NTPDases (apyrases), can hydrolyze ADP to AMP + Pi as efficiently as ATP to ADP + Pi (Plesner, 1995; Kirley, 1997; Meyer-Fernandes et al., 1997). The NTPDases have been characterized from various cell types, including animals, plants, insects and microorganisms. In the latter, different roles were attributed to these enzymes. In yeast, NTPDases are involved in nucleotide sugar transport into the Golgi apparatus and subsequent protein glycosylation (Sansom, 2012). In Legionella pneumophila, secreted NTPDases function as virulence factors (Sansom, 2012). In parasitic protozoa, NTPDases are involved in the infectivity and virulence, as previous reported to *Toxoplasma gondii* (Asai and Suzuki, 1990; Bermudes et al., 1994; Asai et al., 1995; Nakkar et al., 1998), Entameba histolytica (Bakker-Grunwald and Parduhn, 1993; Barros et al., 2000), Tritrichomonas fetus (De Jesus et al., 2002), Leishmania (Leishmania) amazonensis (Berredo-Pinho et al., 2001; Ennes-Vidal et al., 2011) *T. cruzi* (Bernardes et al., 2000; Bisaggio et al., 2003; Meyer-Fernandes et al., 2004) and others. Most of these studies performed the biochemical characterization of the ecto-nucleotidases/NTPDases activity and investigated the role of the ecto-NTPDases in the parasite–host interaction, using enzyme inhibitors and/or polyclonal antibodies. Nevertheless, this approach could result in a poor discrimination between the role of each ecto-enzyme to the parasites infectivity, mainly because these enzymes share close sequence homology (Handa and Guidotti, 1996; Robson et al., 2006). Thus, a conclusive genetic evidence for the correlation between TcNTPDase-1 and *T. cruzi* infectivity and virulence is still missing.

In this study, we use an homologous recombination approach to knockdown or overexpress the TcNTPDase-1 gene, and evaluate the *in vitro* parasite–host cell interaction. TcNTPDase-1 (AY540630.1) corresponds to a single-copy gene (Fietto et al., 2004) that presents different levels of expression according to the evolutive form of *T. cruzi* and to strain/clones from different DTUs (Pinheiro et al., 2006). Following our methodology, we were able to generate viable hemi-knockouts for each allele of TcNTPDase-1 gene from the *T. cruzi* Dm28c strain, with a decrease in TcNTPDase-1 expression at mRNA and protein levels, and similar growth rate in comparison to the WT, except for one clone. Noteworthy, when the TcNTPDase-1 knockout (null mutant) was generated, we could not obtain viable epimastigotes in cultivation. This is an evidence that TcNTPDase-1 act as an essential gene for *T. cruzi*, probably due to its role at the purine salvage pathway. In contrast, in the study from Sansom et al. (2014), they could generate null mutants for the LmNTPDase1 or LmNTPDase2 genes in Leishmania major, showing different contributions of each gene to parasite infectivity. Probably the generation of null mutants was possible since L. major genome contains two putative NTPDase genes, while *T. cruzi* genome contains only one.

Previously, our group have shown that TcNTPDase-1 gene expression increases after *T. cruzi* metacyclogenesis (Silva-Gomes et al., 2014). The trypomastigote forms present TcNTPDase-1 mRNA levels around eight times higher than epimastigotes. In the present study, the same pattern was observed. Interestingly, even presenting the same increasing in the TcNTPDase-1 gene expression when compared to the epimastigote form, the trypomastigote hemi-knockouts keep the TcNTPDase-1 gene expression 50% lower than the trypomastigote WT, similar to observed between epimastigotes. This finding suggest that both TcNTPDase-1 alleles have the same contribution to the *T. cruzi* metacyclogenesis and the silencing of one allele does not impair the increasing in TcNTPDase-1 expression observed after the epimastigote–trypomastigote transition.

There are previous reports about the contribution of ecto-enzymes to the parasite–host cell interaction (Bisaggio et al., 2003; Pinheiro et al., 2006; De Souza et al., 2010; Ennes-Vidal et al., 2011; Mariotini-Moura et al., 2014; Peres et al., 2018). In those studies, the use of Adenosine, polyclonal anti-NTPDase antibodies and ecto-ATPase inhibitors (DIDS, Suramin and CrATP) decrease the parasite–host cell association (adhesion and/or internalization). For instance, pre-incubation of L. amazonensis promastigotes with CrATP decreased both adhesion and internalization of parasite to murine macrophages. As CrATP is a reversible inhibitor of ecto-ATPase activity, removal of CrATP after the pre-incubation with promastigotes recovered the same levels of adhesion and internalization of the untreated parasite. It suggests a key role of ecto-ATPases/ecto-NTPases at the parasite infectivity, but without discriminate the contribution of each enzyme. Herein, the silencing of TcNTPDase-1 gene expression clearly affects the *T. cruzi*-VERO cells interaction, significantly decreasing the adhesion and endocytic indexes, as well as the parasitic load at infected cells. Remarkably, we also could generate, for the first time, a recombinant parasite overexpressing a NTPDase gene from WT or hemi-knockout *T. cruzi*. TcNTPDase-1 overexpression increased by three times the parasite infectivity. In addition, the overexpression from the hemi-knockout parasites could fully recover the *T. cruzi* infectivity and parasitic load at infected cells, proving that the effects observed were specific to TcNTPDase-1.

Taking together, our results support the hypothesis that TcNTPDase-1 has a critical role in *T. cruzi* infectivity, contributing to the adhesion and internalization of the parasite to the mammalian cell, without interferes at the parasite metacyclogenesis. Using murine models, *in vivo* studies are being conducted meaning to investigate the effect of TcNTPDase-1 knockout/overexpression to *T. cruzi* virulence. According to that, we seek to contribute to the evaluation of this enzyme as a promising target for Chagas disease chemotherapy and gene therapy.

## Data Availability Statement

The datasets generated for this study are available on request to the corresponding author.

## Ethics Statement

The experiments with the recombinant *T. cruzi* Dm28c strain were performed in accordance with the recommendations of the Internal Biosafety Commission from the Oswaldo Cruz Institute (IOC/Fiocruz). This study was approved by the National Technical Commission on Biosafety (CTNBio, Brazil), under protocol number 6.145/2018 (Process number: 01250.040532/2018-38).

## Author Contributions

NS-G performed the majority of the experiments, analyzed datasets, and wrote the manuscript. SF, RR, CM, MK, and CS helped with the construction of the knockout and *T. cruzi* overexpressing TcNTPDase-1 gene. EV and GP helped to performing the western blot assays. OM and SF were responsible for experimental design and data analysis. OM was responsible for the final manuscript revision.

## Conflict of Interest

The authors declare that the research was conducted in the absence of any commercial or financial relationships that could be construed as a potential conflict of interest.

## Supplementary Material

FIGURE S1 Strategy and validation for the knocking down TcNTPDase-1 gene in *T. cruzi*. **(A,C)** Diagrams showing the regions used for the construction of the HYGRO and NEO integration cassettes. **(B,D)** Confirmation of integration of the HYG and NEO cassettes into the NTPDase-1 gene locus. Agarose gel analysis of the fragments amplified with the primers (1) HYG_F and HYG_R/(1) NEO_F and NEO_R, respectively. (2) EXTPDASE_F2 and ENO/KAP_R; (3) EXTPDASE_F3 and ENO/KAP_R; (4) GAPDH1/2_F and EXTPDASE_REV2; (5) GAPDH1/2 and EXTPDASE_REV3; (6) Molecular markers (1 kb plus DNA ladder).

FIGURE S2 Standard curve for the absolute quantification of parasitic load in VERO cells infected with TcNTPDase-1 hemi-knockouts. The coefficient of determination (r^2^), PCR efficiency and linear equation are showed at the graph.
