# Supplementary figures and images for: Knocking Down TcNTPDase-1 Gene Reduces in vitro Infectivity of Trypanosoma cruzi
Source: Front Microbiol. 2020 Mar 18;11:434. doi: 10.3389/fmicb.2020.00434 (PMC7094052; doi:10.3389/fmicb.2020.00434)

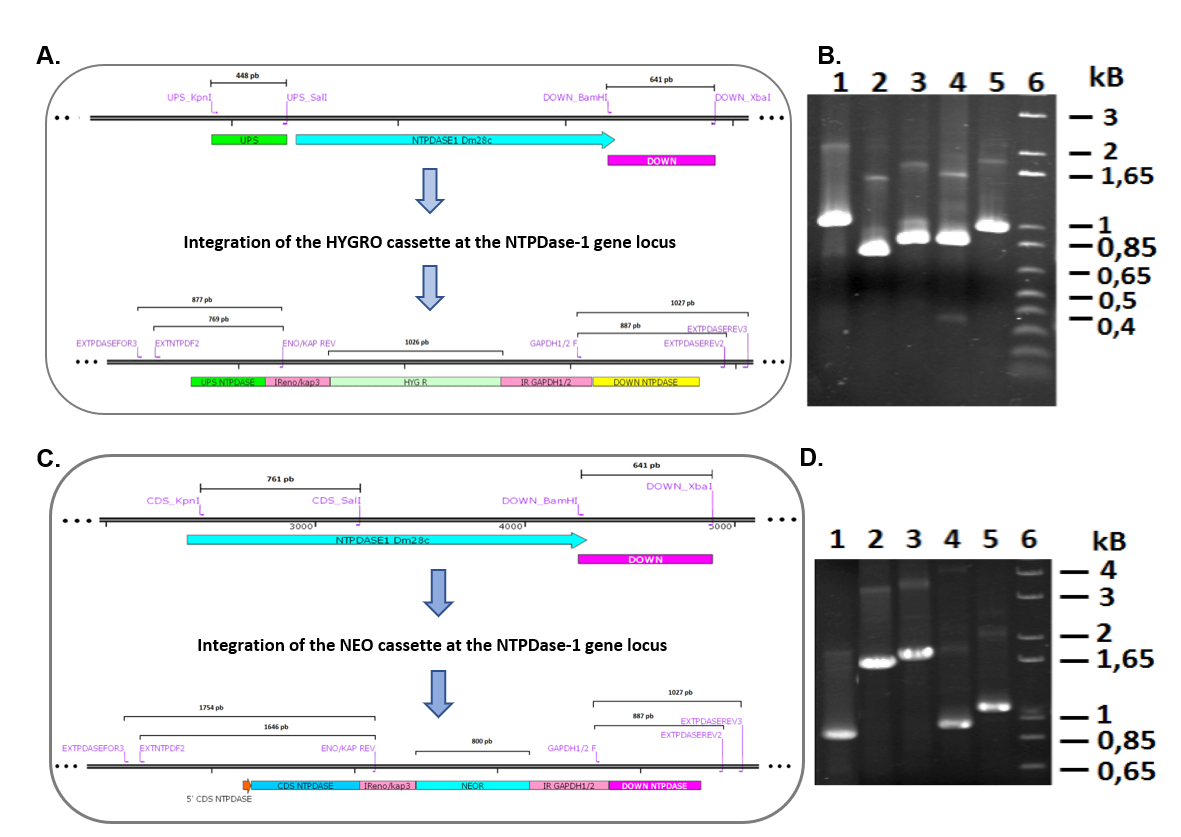

Supplement: FIGURE S1 — Strategy and validation for the knocking down TcNTPDase-1 gene in T. cruzi. (A,C) Diagrams showing the regions used for the construction of the HYGRO and NEO integration cassettes. (B,D) Confirmation of integration of the HYG and NEO cassettes into the NTPDase-1 gene locus. Agarose gel analysis of the fragments amplified with the primers (1) HYG_F and HYG_R/(1) NEO_F and NEO_R, respectively. (2) EXTPDASE_F2 and ENO/KAP_R; (3) EXTPDASE_F3 and ENO/KAP_R; (4) GAPDH1/2_F and EXTPDASE_REV2; (5) GAPDH1/2 and EXTPDASE_REV3; (6) Molecular markers (1 kb plus DNA ladder). [file Image_1.TIF]

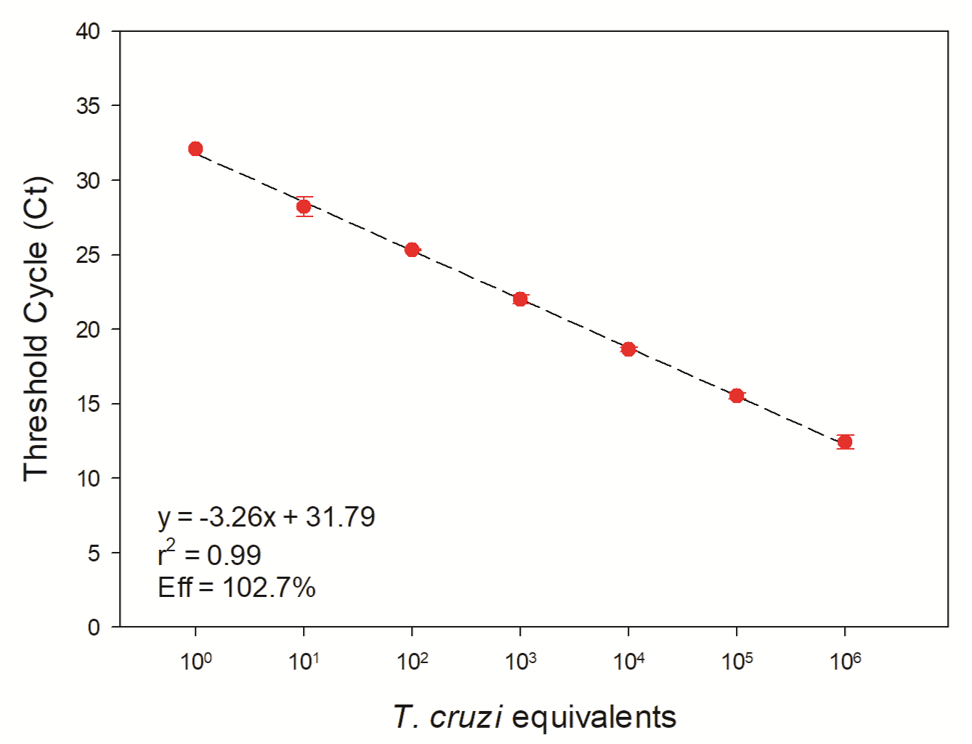

Supplement: FIGURE S2 — Standard curve for the absolute quantification of parasitic load in VERO cells infected with TcNTPDase-1 hemi-knockouts. The coefficient of determination (r2), PCR efficiency and linear equation are showed at the graph. [file Image_2.TIF]
